# Poster Session I - A39 CHARACTERIZING THE ROLE OF STING IN THE INTESTINAL EPITHELIUM

**DOI:** 10.1093/jcag/gwaf042.039

**Published:** 2026-02-13

**Authors:** J J Meade, O Singh, M Nissan, S Girardin

**Affiliations:** Laboratory Medicine and Pathobiology, University of Toronto, Toronto, ON, Canada; Laboratory Medicine and Pathobiology, University of Toronto, Toronto, ON, Canada; University of Toronto Temerty Faculty of Medicine, Toronto, ON, Canada; Laboratory Medicine and Pathobiology, University of Toronto, Toronto, ON, Canada

## Abstract

**Background:**

The intestinal epithelium is a single-cell layer that both absorbs nutrients and provides a critical barrier against pathogenic microbes. Dysregulation of epithelial defense pathways has been implicated in the pathogenesis of inflammatory bowel disease (IBD). Stimulator of interferon genes (STING) is a cytosolic pattern recognition receptor activated by endogenous and microbial cyclic dinucleotides present in the intestinal lumen. Although STING signaling has been linked to intestinal inflammation and IBD, its specific role in epithelial host defense remains poorly understood.

**Aims:**

We hypothesize that epithelial STING plays a critical role in defending against enteric pathogens. Specifically, we aim to:

1) Determine whether STING is expressed and functional in intestinal epithelial cells.

2) Characterize the epithelial response to STING activation.

**Methods:**

Small intestinal Swiss rolls were prepared from wild-type and STING^-/-^ mice injected intraperitoneally with PBS or αCD3. STING expression and localization were examined by RNA in situ hybridization for *Tmem173* transcripts combined with immunofluorescence staining. Ileal organoids were generated from wild-type and STING^-/-^ mice, stimulated with recombinant IFNg, and STING localization was assessed by immunofluorescence. STING signaling was activated in ileal organoids using DMXAA, and downstream pathway activation was evaluated by qPCR and western blotting for canonical STING/IRF3 pathway targets. Global transcriptomic responses were analyzed by bulk RNA sequencing at 24 and 48 hours post-stimulation. All experiments were performed with three biological replicates.

**Results:**

STING was expressed in the intestinal epithelium, and its expression increased following IFNγ exposure both *in vivo* and *de novo*. STING activation in ileal organoids induced IRF3 activation, type III interferon (IFNλ) production, and upregulation of interferon-stimulated genes. Transcriptomic and qPCR analyses revealed enrichment of gene signatures associated with cysteine import and redox regulation, alongside reduced expression of stemness-associated genes. Notably, these changes were not abrogated by TBK1 inhibition, suggesting a TBK1-independent pathway.

**Conclusions:**

These findings identify a previously unrecognized role for epithelial STING in intestinal host defense. Unlike innate immune cells, STING activation in epithelial cells drives a type III interferon response that may enhance barrier immunity. Additionally, STING appears to regulate epithelial redox balance and stem cell proliferation independently of interferon signaling, potentially linking microbial sensing to epithelial homeostasis.

**Funding Agencies:**

CIHR

